# IL-15 Augments TCR-Induced CD4^+^ T Cell Expansion In Vitro by Inhibiting the Suppressive Function of CD25^High^ CD4^+^ T Cells

**DOI:** 10.1371/journal.pone.0045299

**Published:** 2012-09-20

**Authors:** Tom L. Van Belle, Hans Dooms, Tom Boonefaes, Xiao-Qing Wei, Georges Leclercq, Johan Grooten

**Affiliations:** 1 Laboratory of Molecular Immunology, Department of Biomedical Molecular Biology, Ghent University, Ghent, Belgium; 2 Arthritis Center/Rheumatology Section, Boston University School of Medicine, Boston, Massachusetts, United States of America; 3 Tissue Engineering and Reparative Dentistry, Dental School, Cardiff University, Heath Park, Cardiff, United Kingdom; 4 Department of Clinical Chemistry, Microbiology and Immunology, Ghent University, Ghent, Belgium; Institut Pasteur, France

## Abstract

Due to its critical role in NK cell differentiation and CD8^+^ T cell homeostasis, the importance of IL-15 is more firmly established for cytolytic effectors of the immune system than for CD4^+^ T cells. The increased levels of IL-15 found in several CD4^+^ T cell-driven (auto-) immune diseases prompted us to examine how IL-15 influences murine CD4^+^ T cell responses to low dose TCR-stimulation *in vitro*. We show that IL-15 exerts growth factor activity on both CD4^+^ and CD8^+^ T cells in a TCR-dependent and Cyclosporin A-sensitive manner. In CD4^+^ T cells, IL-15 augmented initial IL-2-dependent expansion and once IL-15Rα was upregulated, IL-15 sustained the TCR-induced expression of IL-2/15Rβ, supporting proliferation independently of secreted IL-2. Moreover, IL-15 counteracts CD4^+^ T cell suppression by a gradually expanding CD25^High^CD4^+^ T cell subset that expresses Foxp3 and originates from CD4^+^CD25^+^ Tregs. These *in vitro* data suggest that IL-15 may dramatically strengthen the T cell response to suboptimal TCR-triggering by overcoming an activation threshold set by Treg that might create a risk for autoimmune pathology.

## Introduction

The pro-inflammatory cytokine IL-15 is produced predominantly by activated monocytes/macrophages [1], but can also be produced by T cells [2] and in mucosal tissues by epithelial cells [3]–[5]. Expressed by dendritic cells in response to type I IFN, double-stranded RNA, or lipopolysaccharide [6], IL- is involved in several types of infections [7]. Bacterial infection with *Salmonella* or *Mycobacterium* [8], [9], or viral infection with *Influenza* has been shown to induce the expression of IL-15 [10], [11]. IL-15 is also implicated in several inflammatory disorders and autoimmune diseases [1], [12]–[14]. IL-15 is present at high concentrations in rheumatoid arthritis synovial fluid [15], [16] and elevated in the serum of patients with systemic lupus erythematosus [17] or type 1 diabetes [18]. Additionally, IL-15 is heightened in the affected tissues in autoimmune thyroid disease [19] and celiac disease [20].

IL-15 given exogenously can enhance immune responses and these properties are exploited in tumor therapy [21], [22] and in vaccination strategies [23]–[27]. IL-15 helps the development and activity of NK cells [28], CD4^+^ T cells [29], [30] and CD8^+^ T cells [31]–[33]. For instance, IL-15 prolongs the survival of effector T cells against *Listeria monocytogenes* and *Mycobacterium bovis* [10] and augments the response by respiratory CD8^+^ T cells in *Influenza* infections [11]. IL-15 also augments the generation of tetanus toxoid-specific effector CD4^+^ T cells in rhesus macaques [34]. Our previous studies have shown that IL-15 enhances the proliferative response of TCR-stimulated CD4^+^ T cells *in vitro* [29], [35]. IL-15 treatment is known to promote a persistent immune response through its actions on memory CD8^+^ T cells [32], [36], [37] and the proliferation of human memory CD4^+^ T cells *in vitro* and mouse Ag-specific CD4^+^ memory T cells in vivo [38], [39]. Initial studies suggested that IL-15 is mainly critical for CD8^+^ T cell homeostasis [40]–[42], and less for homeostasis of naive or memory CD4^+^ T cells [5], [43]–[45], especially because normal numbers of memory-phenotype CD4^+^ T cells are present in IL-15–deficient mice [46]. Similarly, IL-15 is reported to have only a minimal role in the homeostasis of Ag-specific CD4^+^ memory T cells [47]. However, recent studies have revealed that IL-15 is important for the homeostatic proliferation of both types of memory cells [48]–[50]. For instance, in normal nonlymphopenic hosts where IL-7 levels are low, virus Ag-specific CD4^+^ memory cells are dependent on IL-15 for their basal homeostatic proliferation and long-term survival [49]. Also, the IFN-γ-producing memory CD4^+^ T cells induced by transient bacterial infection with Listeria monocytogenes express IL-15Rβ and are responsive to IL-15 [50].

Deficient development or function of CD25^+^CD4^+^regulatory T (Treg) cells causes organ-specific autoimmune diseases in animal models, demonstrating their crucial role in maintaining self-tolerance [51]–[54]. In human peripheral blood, approximately 1.5–3.0% of total CD4^+^ T cells express high levels of CD25 [55] and have similar regulatory properties as murine CD25^+^CD4^+^ T cells [55]–[57]. Potent TCR stimulation [58], [59], but also cytokines, e.g. high dose IL-2 [60]–[62] or IL-6 [63] render effector T cells resistant to the suppression by Treg cells. The role of IL-15 in the homeostasis and function of Treg cells is not clear, but IL-15 can partially support Treg cell development in the absence of IL-2 [64] and protect human effector T cells against Treg cell action [65].

In this study we examined how low doses of IL-15 influence a primary CD4^+^ T cell response to low doses of TCR/CD3-triggering. The growth factor activity of IL-15 on CD4^+^ T cells depends on TCR-induced IL-2 production in the first phase of activation, and only later supports CD4^+^ T cell expansion independent of IL-2. IL-15 also promotes CD4^+^ T cell expansion in a more indirect way, namely by lifting the suppressive activity Foxp3 expressing CD25^High^ CD4^+^ T cells that originate from natural CD25^+^CD4^+^ Treg cells after TCR-stimulation.

## Results

### IL-15 not only Exerts Growth Factor Activity on CD8^+^ T Cells, but also on CD4^+^ T Cells

**Figure 1:**
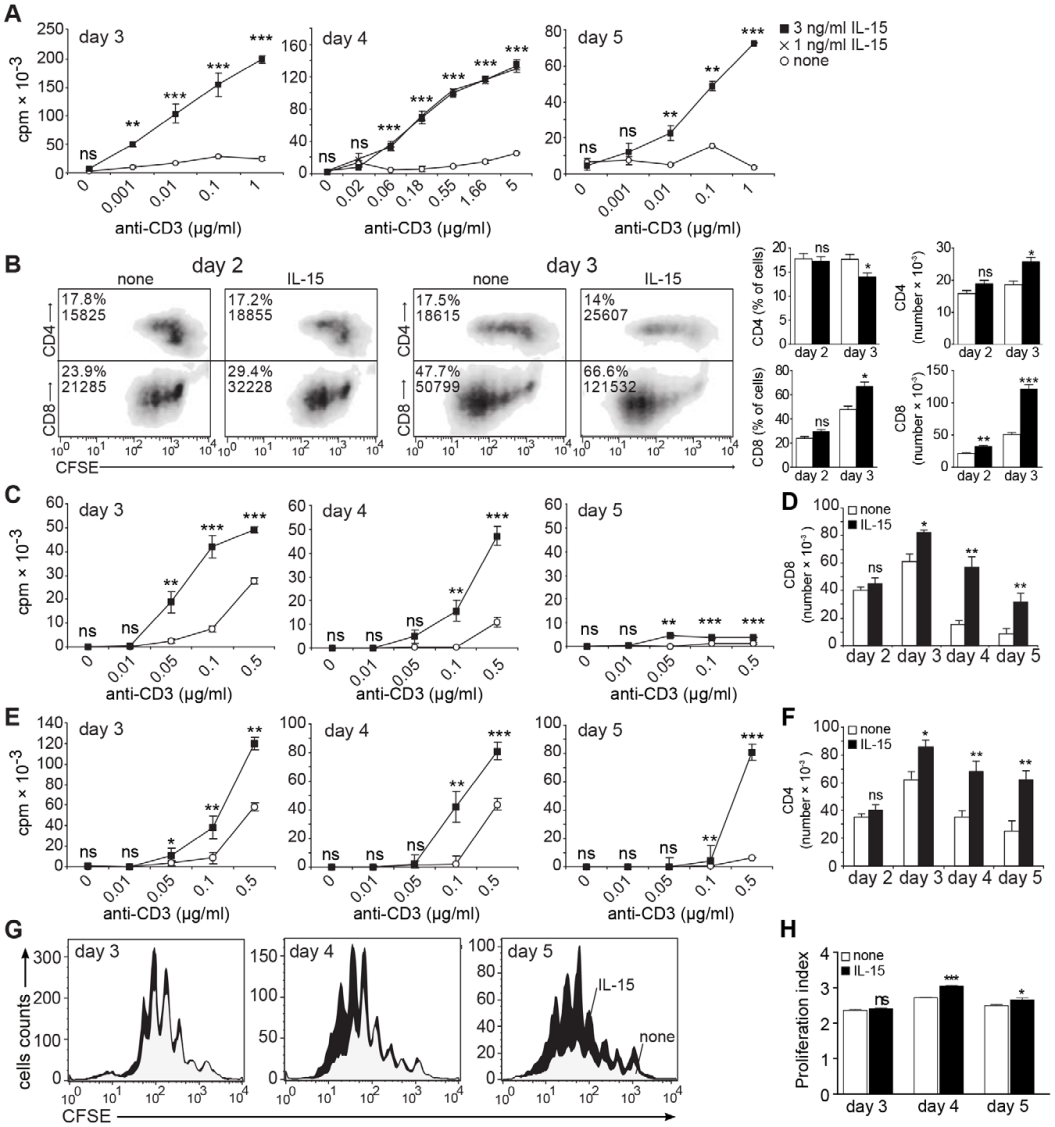
IL-15 enhances the strength and duration of TCR/CD3-triggered proliferation by CD8^+^ and CD4^+^ T cells. (**A**) Bulk splenocytes were stimulated with soluble anti-CD3 mAb and ^3^H-thymidine incorporation was measured (white circles: none; crosses: 1 ng/ml IL-15; black squares: 3 ng/ml IL-15). (**B**) CFSE-labeled splenocytes were stimulated using 0.1 µg/ml anti-CD3 mAb and supplemented or not with 3 ng/ml rhIL-15. Cells were stained for CD8 or CD4 after 2–3 days. Shown are representative density plots with inset values reflecting percentage and absolute numbers of CD4^+^ or CD8^+^ T cells in the viable cell gate and summarizing bar graphs. (**C–F**) CD8^+^ T cells (***C,D***) or CD4+ T cells (***E,F***) were stimulated with soluble anti-CD3 mAb and irradiated TdACs in the absence or presence of 3 ng/ml rhIL-15. ^3^H-thymidine incorporation (***C,E*** *;* white circles: none, black squares: 3 ng/ml IL-15) or cell numbers (***D,F***; 0.1 µg/ml anti-CD3; white bars: none, black bars: 3 ng/ml IL-15) were measured. (**G**) CFSE-labeled CD4^+^ T cells were stimulated as in *F*. Shown are CFSE histogram overlays, gated on viable CD4^+^ T cells, of cultures without (grey) or with (black) exogenous rhIL-15. The heights of the profiles were normalized to the absolute viable CD4^+^ T cell numbers present in the culture, as determined by flow cytometry using fluorescent micro-beads as a reference. (**H**) Proliferation index of CD4^+^ T cells in cultures without (white squares) or with (black squares) supplemented IL-15 (3 ng/ml). For all panels, data are from triplicate wells in one representative of four repeat experiments. **A, C**: Symbols in line graphs reflect mean ± SD. **D, F, H**: Bar graphs represent the mean±SEM. In all relevant panels, statistical significance between IL-15-supplemented and non-supplemented cultures was calculated per anti-CD3 concentration using student t-test: ns = not significant, * *P*<0.05, ** *P*<0.01, *** *P*<0.001. All experiments were performed at least 3 times with similar results.

Anti-CD3 stimulation caused a dose dependent proliferative response of C57Bl/6 splenocytes and supplementation with IL-15, even at doses as low as 1 ng/ml, markedly increased both the amplitude and the duration of the proliferative response (Figure 1A). This growth enhancing effect of IL-15 was not mouse strain dependent or restricted to cells from a specific secondary lymphoid compartment, as bulk lymph node cells and splenocytes from Balb/c responded similarly (data not shown).

To assess the effect of IL-15 on CD4^+^ and CD8^+^ T cells, we stimulated CFSE-labeled splenocytes with anti-CD3. Dye dilution analysis showed that both CD8^+^ and CD4^+^ T cells proliferate, but that CD8^+^ T cells proliferated faster than CD4^+^ T cells, as evidenced by the higher number of generations distinguished 2 days and especially 3 days after stimulation (Figure 1B). CD8^+^ T cells were also more sensitive to stimulation, as they proliferated in response to lower anti-CD3 concentrations (data not shown). Consequently, the CD8^+^ T cell subset dominated the splenocyte cultures after 3 days of activation (Figure 1B). Nonetheless, absolute CD4^+^ T cell numbers did increase in time, albeit less dramatically than CD8^+^ T cells. Addition of IL-15 increased both CD4^+^ and CD8^+^ T cells number (Figure 1B), suggesting IL-15 growth enhancing effect can target both T cell subsets.

Given the predominant outgrowth of the CD8^+^ subset, we next analyzed the effect of IL-15 on anti-CD3 stimulation of isolated CD4^+^ and CD8^+^ T cells. Exogenous IL-15 boosted and prolonged the proliferative response of both CD8^+^ T cells (Figure 1C,D) and CD4^+^ T cells (Fig. 1*E,F*), an effect dependent on TCR-triggering (Figure 1C,E). We used irradiated T cell-depleted accessory cells (TdAC) to crosslink the anti-CD3 mAb, but IL-15 also increased proliferation and cell numbers during stimulation with plate-bound anti-CD3 and soluble anti-CD28, suggesting IL-15 acted directly on the T cells (data not shown). The doses of IL-15 used did not cause proliferation during stimulation with soluble anti-CD3 in the absence of TdAC or in the absence of stimulation (data not shown). Similar results were obtained using either recombinant human or murine IL-15 (data not shown). CFSE proliferation studies showed that CD4 T cells had a higher proliferation index in IL-15-supplemented cultures (Figure 1G, H), although this parameter is unlikely to explain by itself the augmented cell numbers. It is possible that better cell survival in each generation also contributes to the higher total cell counts. Together, the similarity in magnitude and time course of the responses of the CD4^+^ and CD8^+^ T cell subsets indicates that IL-15 is not a selective growth factor for CD8^+^ T cells, but can also exert this function on CD4^+^ T cells to prolong expansion of TCR-stimulated CD4^+^ T cells.

### IL-15 has an IL-2-dependent Mode of Action on CD4^+^ T Cells, but not on CD8^+^ T cells

**Figure 2:**
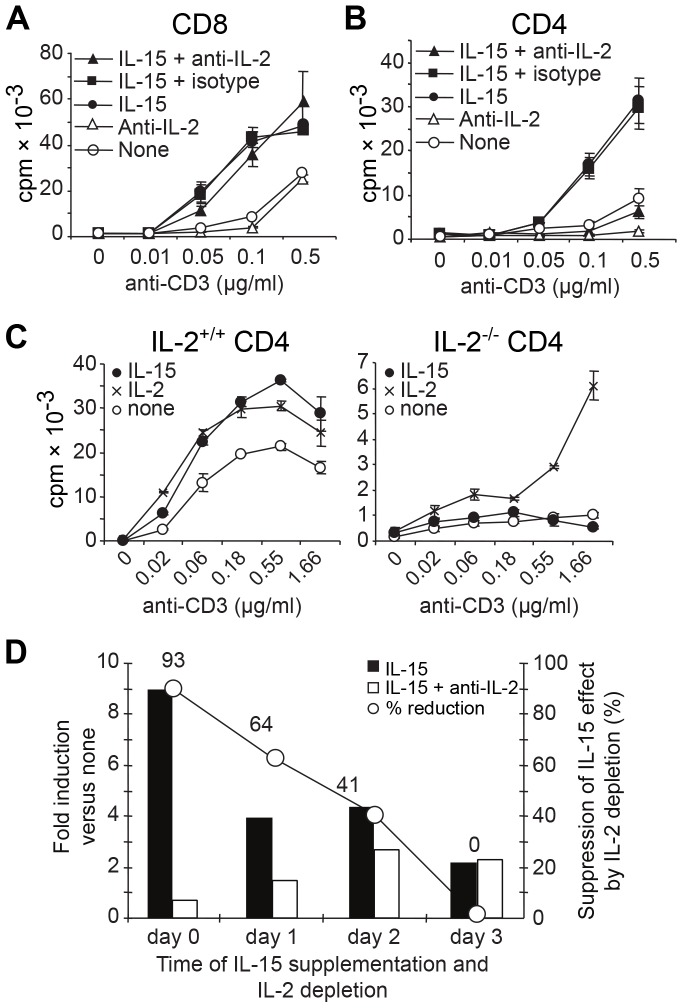
IL-15 growth factor activity depends on TCR-signals, but is IL-2-dependent only in CD4^+^ T cells. (**A**) CD8^+^ or (**B**) CD4^+^ T cells were stimulated with anti-CD3 mAb and irradiated TdACs in the absence or presence of 3 ng/ml rhIL-15, or 10 µg/ml anti-IL-2 mAb (JES6-5H4) or isotype control, as indicated. On day 4, ^3^H-thymidine incorporation was measured. Results are from one of five experiments. (**C**) IL-2^+/+^ or IL-2^−/−^ CD4^+^ cells were stimulated with anti-CD3 mAb and irradiated IL-2^+/+^ and IL-2^−/−^ splenocytes, respectively, in the absence (white circles) or presence of 1 ng/ml IL-2 (crosses) or 3 ng/ml IL-15 (black circles). (**D**) CD4^+^ cells were stimulated for 5 days with 0.1 µg/ml anti-CD3 and irradiated TdACs. On day 0, 1, 2, or 3, IL-15 alone or IL-15 plus anti-IL-2 mAb was added. On day 4, proliferation was measured by ^3^H-thymidine incorporation and represented as bar graphs (with left Y-axis) as ratio of cpm_IL-15_/cpm_none_ (black bars) or cpm_IL-15 plus anti-IL-2_/cpm_none_ (white bars). The line represents the percentage of reduction of the IL-15 effect by IL-2 depletion as calculated using the following formula: [(cpm_IL-15_– cpm_IL-15 plus anti-IL-2_)/cpm_IL-15_]×100. Results are from one representative out of three experiments.

We next determined whether the IL-15 growth factor activity reflects a redundant activity with IL-2 because of shared receptor subunits or a dependence on TCR-induced endogenous IL-2. We found that depletion of IL-2 using neutralizing mAb (clone JES6-5H4: Figure 2A,B; clone S4B6: not shown) blocked the proliferative response by CD8^+^ T cells (Figure 2A) and CD4^+^ (Figure 2B) T cells to low concentrations of anti-CD3. When IL-2 was depleted, IL-15 could support growth of CD8^+^ T cells (Figure 2A), but not of CD4^+^ T cells (Figure 2B). To further corroborate the antibody-mediated depletion of IL-2, we stimulated purified CD4^+^ T cells from IL-2^−/−^ or IL-2^+/+^ mice with anti-CD3 in the presence of IL-2^−/−^ or IL-2^+/+^ APC, respectively. As expected from our data above, both IL-2 and IL-15 increased the dose-dependent response of IL-2^+/+^ CD4^+^ T cells to anti-CD3 stimulation (Figure 2C). In contrast, IL-2^−/−^ CD4^+^ T cells were deficient in proliferation in response to anti-CD3. Addition of low doses IL-2 increased their proliferative response, albeit to a limited extend. The lack of a full restoration may be because the added amount of IL-2 was lower than what is naturally produced [66] or because IL-2^−/−^ T cells contain a larger number of effector/memory T cells [67], which respond differently to IL-2. IL-15 did not increase the anti-CD3-stimulated proliferation of IL-2^−/−^ CD4^+^ T cells at all.

Since sensitivity of CD4^+^ T cells to the growth factor activity of IL-15 depends on TCR-signals, we tested whether also the IL-2 dependence of the IL-15 growth factor activity changes as CD4^+^ T cells become activated. To address this, we simultaneously added IL-15 and depleted IL-2 after 1, 2, or 3 days of TCR-stimulation. This showed that the addition of IL-15 increases CD4^+^ T cell proliferation irrespective of the time of addition, but addition of IL-15 at the start of activation allows IL-15 to deploy its full growth factor potential (Figure 2D). Importantly, IL-2 depletion progressively lost its capacity to dampen IL-15 growth factor activity. As a result, IL-15 exerted its growth promoting effect independently of IL-2 by day 3 of stimulation.

Collectively, these data indicate that IL-15 growth factor activity on CD4^+^ T cells depends on endogenous IL-2 at the start of activation, but not at later time points. On CD8^+^ T cells however, IL-2 and IL-15 exert redundant growth factor activities.

### IL-15-expanded CD4^+^ T cells Remain Dependent on IL-15

**Figure 3:**
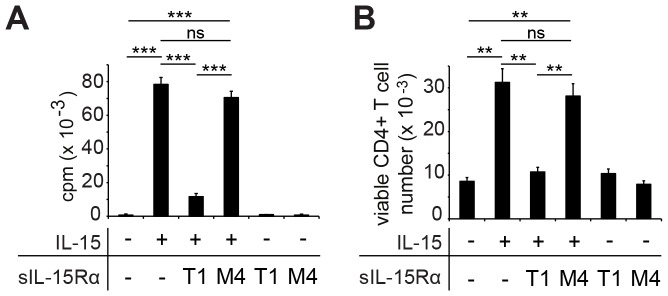
TCR-triggered IL-15-expanded CD4^+^ T cells remain dependent on IL-15 for expansion and survival. CD4^+^ T cells were stimulated with 0.1 µg/ml anti-CD3 and irradiated TdACs without or with 3 ng/ml IL-15. After 3 days, 50 ng/ml soluble IL-15Rα (sIL-15Rα, T1, 50 ng/ml) or control mutated sIL-15Rα (M4), lacking IL-15 binding capacity, were added. (**A**) Proliferation of CD4^+^ T cells, as determined by ^3^H-thymidine incorporation of triplicate cultures on day 5. (**B**) Viable CD4^+^ cell recovery on day 5, determined by flow cytometry. Bar graphs represent the mean±SEM. Statistical significance was calculated using student t-test: ns = not significant, ** *P*<0.01, *** *P*<0.001. Data are representative of three independent experiments.

A previous report indicated that IL-15Rα is a negative regulator of TCR-stimulated proliferation of CD4^+^ T cells [68]. If so, blocking IL-15 should enhance proliferation of TCR-stimulated CD4^+^ T cells. To test this, we added a soluble form of the IL-15Rα protein (sIL-15Rα; T1) to scavenge IL-15 [69] on day 3 of stimulation. Viable CD4^+^ T cell counts (Figure 3A) and ^3^H-thymidine incorporation (Figure 3B) showed that administration of sIL-15Rα completely abrogated the IL-15-induced increase in CD4^+^ T cell numbers. Addition of the control protein M4, lacking the critical IL-15 binding disulfide bonds in the Sushi domain [70], did not affect the CD4^+^ cell response to IL-15. So, IL-15 does not reprogram CD4^+^ T cells for self-sustained growth. Rather, after activation CD4^+^ T cells depend on IL-15 to maintain increased cell numbers and proliferation.

### IL-15 Supports Prolonged Cycling of CD25^Intermediate^CD4^+^ T cells in the Presence of CD25^High^CD4^+^ T Cells

**Figure 4:**
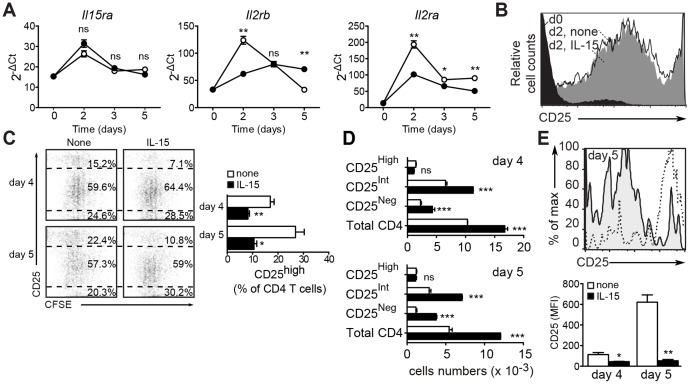
Exogenous IL-15 decreases the fraction but not the absolute number of CD25^High^ CD4^+^ T cells. (**A**) IL-15 alters TCR-induced gene expression of IL-2 and IL-15 receptor subunits. Purified CD4+ T cells were stimulated with 0.1 µg/ml anti-CD3 mAb and irradiated TdACs in the absence (white circles) or presence (black circles) of 3 ng/ml IL-15. At the indicated time points, CD4^+^ T cells were purified for PCR analysis of indicated gene transcripts and expressed as 2^−ΔCt^ where ΔCt = Ct_target gene_ - mean Ct_normalization genes_. Statistical significance was calculated using student t-test: ns = not significant, ** *P*<0.01, *** *P*<0.001. (**B**) Spleen CD4^+^ T cells were left unstimulated or stimulated with anti-CD3 in the presence or absence of IL-15 for two days, as indicated. Shown are histogram overlays of CD25 expression, gated on viable CD4^+^ T cells (d: day). (**C**) CFSE-labeled CD4^+^ T cells were stimulated with 0.1 µg/ml anti-CD3 and irradiated TdAC, without or with 3 ng/ml IL-15 for four or five days. Shown are flow cytometry dot plots of CFSE dye dilution versus CD25 expression in the viable CD4^+^ gate (left, shown percentages were calculated on viable CD4^+^ T cells) and summarizing bar graph of the CD25 high fraction (left). Statistical significance was calculated using student t-test: * *P*<0.05, ** *P*<0.01. (**D**) Absolute numbers of viable CD25^High^, CD25^Int^, CD25^Negative^ and total CD4^+^ T cells, as determined by flow cytometry using micro-beads as reference. White and black bars represent stimulation with anti-CD3 in the absence or presence of 3 ng/ml rhIL-15, respectively. Statistical analysis was calculated using 2-way ANOVA and Bonferroni posttest: ns = not significant, *** *P*<0.001. Data shown are representative of three independent experiments. (**E**) CFSE-labeled CD4^+^ T cells were stimulated as in *B*. Shown are histogram overlays (top) of CD25 expression of the last generation of viable CD4^+^ T cells (by CFSE dilution) on day 5 after stimulation in the absence (dashed line) or presence (shaded) of 3 ng/ml rmIL-15. Bar graph (bottom) represents the mean fluorescenceIntensity of CD25 expression in the last generation, as shown in the overlays. Statistical significance was calculated using student t-test: * *P*<0.05, ** *P*<0.01.

We next assessed the gene expression of the IL-15 receptor subunits after anti-CD3 stimulation of CD4^+^ T cells. The mRNA levels of IL-15Rα and IL-2/15Rβ (CD122) peaked after two days by two- and four-fold, respectively (Figure 4A). Exogenous IL-15 did not affect the activation-induced expression of IL-15Rα. However, IL-15 attenuated the initial upregulation of IL-2/15Rβ (approximately two-fold) and maintained this expression level throughout the culture period. As a result, IL-15Rβ is expressed two-fold higher on day 5 when compared with unstimulated CD4^+^ T cells or day 5 control-stimulated CD4^+^ T cell cultures (Figure 4A). Flow cytometry showed that expression of CD122 was increased in the entire CD4^+^ T cell population, rather than reflecting the outgrowth of a specific subset (data not shown).

On the other hand, IL-15 supplementation limited the anti-CD3-induced upregulation of IL-2Rα (CD25)(Figure 4A), an activation marker known to confer sensitivity to AICD, but also a Treg marker [51], [54]. This could reflect a decrease in surface expression of CD25 on all activated T cells or a reduction in the fraction of CD25^+^ Treg cells. To test this, we stimulated CFSE-labeled CD4^+^ T cells to monitor CD25 expression. In line with its transcript expression, CD25 membrane expression was strongly upregulated upon TCR stimulation in both control and IL-15 supplemented cultures (Figure 4B).Interestingly, low-dose anti-CD3 stimulation generated two CD25-positive populations, expressing either high or intermediate (Int) levels of CD25. On day two, the effect of IL-15 supplementation on CD25 at the mRNA level had not yet translated to the level of surface protein (Figure 4B). CFSE-dilution experiments showed that both CD25^Int^ and CD25^High^ subsets actively proliferated (Figure 4C, left panels). The CD25^High^ subset constituted approximately 15% and 22% of the viable CD4^+^ T cell population after four and five days, respectively. Supplementation with human IL-15 reduced the relative proportion of CD25^High^ cells to approximately 7% and 11% (Figure 4C, right panels), seemingly sustaining mainly the expansion of the CD25^Int^ subset. A similar trend was also observed using murine IL-15 (data not shown). Exogenous IL-15 did increase the absolute numbers of CD4^+^ T cells expressing no or intermediate levels of CD25, but not those with high levels of CD25, both on day four and five (Figure 4D). This indicates that the drop in the CD25^High^ CD4^+^ T cells is relative and results from a greater expansion of cells expressing no or intermediate levels of CD25. The cells that had undergone most divisions upon stimulation with anti-CD3 in the absence of IL-15 expressed mostly high levels of CD25 (Figure 4E). In contrast, in the presence of exogenous IL-15, the cells that had undergone most divisions mostly expressed intermediate levels of CD25. Thus, low levels of TCR stimulation gradually expanded a CD4^+^ T cell subset expressing high levels of CD25. Exogenous IL-15 on the other hand, extended the proliferative response by supporting the expansion of the CD25^Int^ cell subset in the presence of CD25^High^CD4^+^ T cells.

### CD25^High^CD4^+^ T cells Originate from Naturally Occurring CD25^+^CD4^+^ Regulatory T cells and Inhibit Proliferation of CD25^−/low^ CD4^+^ T cells

**Figure 5:**
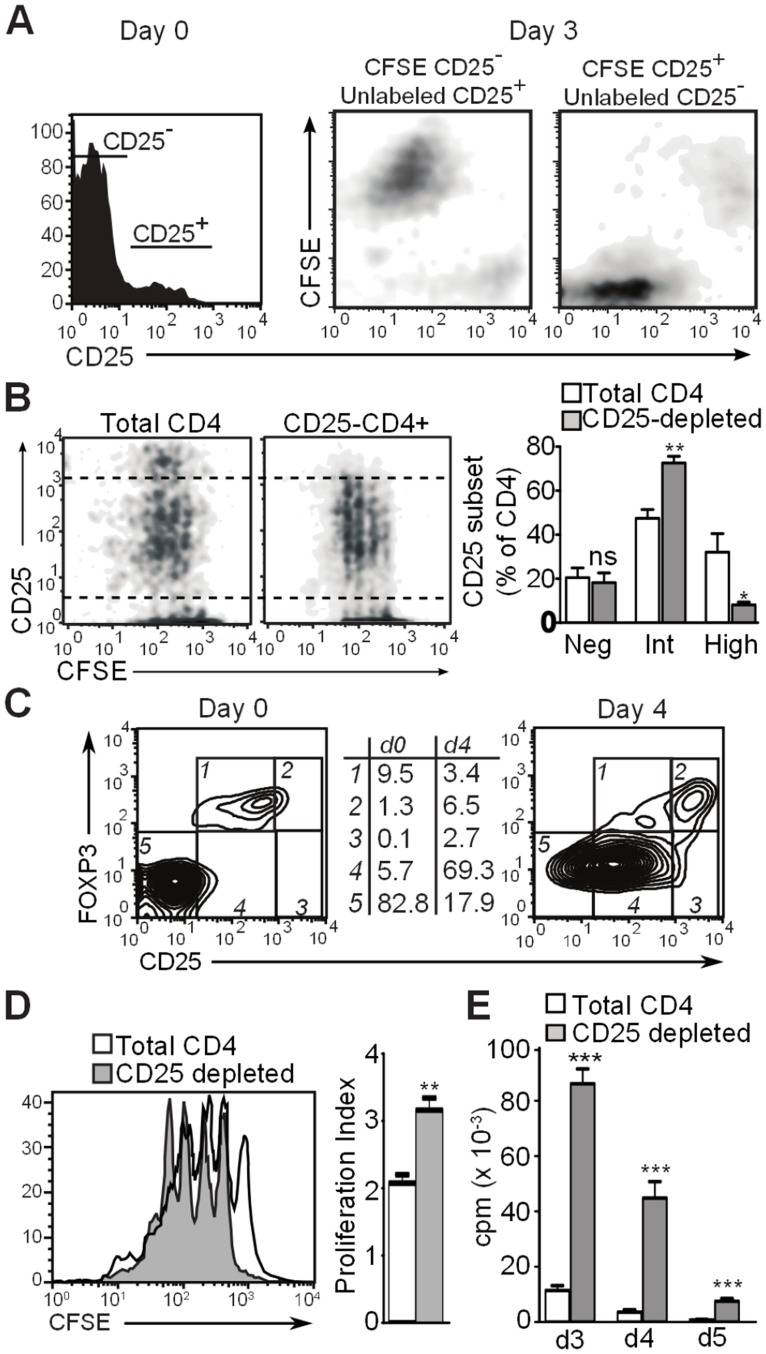
CD25^High^ CD4^+^ T cells originate from natural CD25^+^ CD4^+^ T cells. (**A**) Histogram showing CD25 expression pattern of unstimulated CD4^+^ T cells (day 0; top). Isolated CD25^−^ and CD25^+^ CD4^+^ T cells were co-cultured in a cross-over setup with only one of the subsets carrying CFSE label. Three days after stimulation with 0.1 µg/ml anti-CD3 mAb and irradiated TdAC, CD25 expression and CFSE dye dilution were measured using flow cytometry and displayed as density plots (bottom). (**B**) CFSE-labeled total CD4^+^ T cells (top) or CD25^+^-depleted CD4^+^ T cells (bottom) were stimulated as in (**A**). Shown are flow cytometry density plots of CFSE dye dilution versus CD25 expression gated on viable CD4^+^ T cells. Percentages indicate the percentage of CD25^High^ or CD25^Int^ cells within all CD25^+^ positive CD4^+^ T cells. (**C**) Contour plots showing CD25 versus Foxp3 expression of unstimulated and day 4-stimulated CD4^+^ T cells. Numbers on the right reflect the percentages within the gates 1–5, gated on viable CD4^+^ T cells. (**D**) CFSE-labeled total CD4^+^ T cells (shaded) or CD25^+^-depleted CD4^+^ T cells (solid line) were stimulated with anti-CD3 (0.1 µg/ml) and irradiated TdACs for 3 days. Shown are histogram overlays gated on viable CD4^+^ T cells and bar graph of proliferation index mean±SEM. (**E**) Total CD4^+^ T cells or CD25-depleted CD4^+^ T cells were stimulated with anti-CD3 (0.1 µg/ml) and irradiated TdACs for indicated time and proliferation was measured via ^3^H-thymidine incorporation. (**B, D, E**) Bar graphs represent the mean±SEM. Statistical significance was calculated by student t-test, ns: not significant, * *P*<0.05, ** *P*<0.01, *** *P*<0.001. Experiments performed at least 3 times with similar results.

We hypothesized that the CD25^High^CD4^+^ T cell subset originates from naturally occurring CD25^+^CD4^+^ T cells that persist during TCR stimulation as a distinct CD25^High^ population capable of controlling the proliferative response of conventional CD25^−^CD4^+^ T cells. To verify this, we first did a criss-cross experiment in which we co-cultured CFSE-labeled CD25 negative CD4 cells with unlabeled CD25^+^ CD4^+^ T cells, and vice versa, at the normal CD25^+^:CD25^−^ ratio. In both instances, a CD25^Int^ and CD25^High^ population was generated. The CD25^Int^ population only contained CFSE when the CD25^−^CD4^+^ T cells were CFSE-labeled at the start of culture (Figure 5A). Conversely, the CD25^High^ population only contained CFSE when the CD25^+^ CD4^+^ T cell subset was CFSE-labeled. Both the CD25^Int^ and the CD25^High^ populations were cycling, thus confirming their proliferative capacity (Figure 5A). Second, we compared CD25 expression levels in anti-CD3-stimulated cultures of total CD4^+^ T cells versus CD25-depleted CD4^+^ T cells and found that initial depletion of CD25^+^ cells reduced the generation of a CD25^High^ subset by 2–3-fold (Figure 5B). Third, the CD25^High^ subset of TCR-stimulated CD4^+^ T cells expresses Foxp3, while the CD25^Int^ subset is predominantly Foxp3^−^ (Figure 5C). Finally, CFSE dye dilution showed augmented cell division of CD25^+^-depleted CD4^+^ T cell cultures as compared with total CD4^+^ T cells (Figure 5D). ^3^H-thymidine incorporation experiments confirmed this stronger proliferation of cultures of CD25^+^-depleted CD4^+^ T cells versus total CD4^+^ T cells (Figure 5E). Combined, these observations indicate that during activation of CD4^+^ T cells, a CD25^High^ CD4^+^ T cell subset develops from the CD25^+^ Treg population.

### IL-15 reduces the Suppressive Capacity of CD25^High^ CD4^+^ T cells

**Figure 6:**
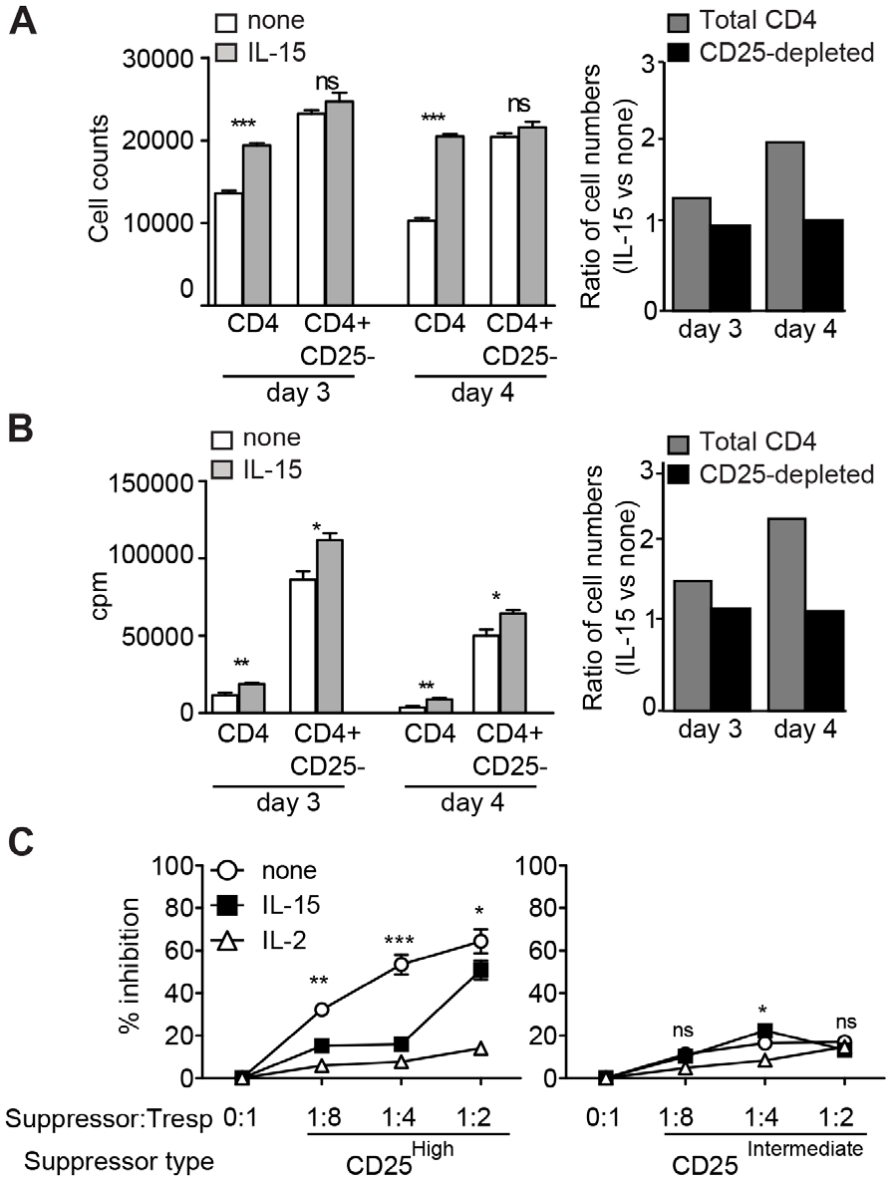
IL-15 blocks suppression by CD25^High^ CD4^+^ T cells. (A) CD4^+^ T cell numbers and (B) proliferation of total CD4^+^ T cells (white bars) or CD25-depleted CD4^+^ T cells (grey bars) stimulated with anti-CD3 mAb, irradiated TdACs and supplemented or not with IL-15. Data are expressed as absolute values (left panels) or fold increase of IL-15-supplemented versus non-supplemented cultures (right panels; 1 reflects no effect of IL-15 addition). Bar graphs represent the mean ± SEM. Statistical significance was calculated by student t-test, ns: not significant, * *P*<0.05, ** *P*<0.01, *** *P*<0.001. (C) CD25^−^ CD4^+^ responder T cells (Tresp) were co-cultured with CD25^High^ (left) or CD25^Int^ (right) CD4^+^ T cells, FACS-purified from activated CD4^+^ T cell cultures as described in Materials and Methods, as putative suppressors at indicated ratios, prior to stimulation with 0.1 µg/ml anti-CD3, irradiated TdACs, and indicated cytokines for 3 days. Proliferation was measured via ^3^H-thymidine incorporation and the percent suppression was calculated as follows: % suppression = 100×(cpm_Responder_ – cpm_Coculture_)/cpm_Responder_. Statistical significance between IL-15-supplemented and non-supplemented cultures was calculated using two-way ANOVA with Bonferroni posttest: ns: not significant, * *P*<0.05, ** *P*<0.01, *** *P*<0.001 (statistical analysis versus IL-2-supplemented cultures not shown). All experiments were performed at least 3 times with similar results.

Because CD25^High^ CD4^+^ T cells originated from CD25^+^ CD4^+^ Treg and maintained Foxp3 expression upon activation, we tested whether CD25^High^ CD4^+^ T cells also maintained suppressive function in the presence or absence of exogenous IL-15. We addressed this by testing whether IL-15 still exerted growth factor activity on CD25-depleted CD4^+^ T cells, i.e. a conventional CD4^+^ T cell population no longer under control of natural Treg. CD25 depletion indeed dramatically enhanced CD4^+^ T cells proliferation (see Figure 5D,E). Strikingly, IL-15 addition to Treg-depleted cultures could no longer increase the absolute cell numbers (Figure 6A) and cell proliferation (Figure 6B). It is possible that IL-15 has no additional effect because IL-2 is less limiting in the absence of Treg, or alternatively, that IL-15 enhances CD4^+^ T cell responses by inhibiting the suppressive capacity of CD25^High^CD4^+^ T cells. We examined the latter using a conventional suppression assay. Conventional CD25^−^ CD4^+^ T cells as responder T cells (Tresp) were co-cultured at different ratios with CD25^High^ or CD25^Int^ CD4^+^ T cells that were isolated after three days of low-dose anti-CD3 stimulation. In line with the differential Foxp3 expression (Figure 5C), CD25^High^ CD4^+^ T cells strongly inhibited Tresp proliferation but CD25^Int^ CD4^+^ T cells did not (Figure 6C, left versus right panel). Similar to IL-2, low-dose IL-15 allowed proliferation of Tresp in the presence of the CD25^High^ Treg fraction at co-culture ratios of 1∶8 to 1∶4. Thus, IL-15 supplementation overcomes Teff suppression by a Foxp3-expressing CD25^High^ CD4^+^ T cell subset that originates from natural Treg after activation.

## Discussion

Because of the common usage of IL-2/15Rβ and γ_C_ receptor components, IL-2 and IL-15 share several biological activities among which the capacity to elicit T cell expansion [1], [71]. In this study, we examined how low doses of exogenous IL-15 enhance proliferative responses to low-level TCR stimulation of T cells. We first found that the growth factor activity of low-dose IL-15 depends on concomitant TCR stimulation, in which case it augments both the strength and the duration of CD4^+^ T cell responses. This is in line with several reports showing that IL-15 does not support bystander proliferation of CD4^+^ T cells [72]–[74]. Conceivably, innate immune danger signals, such IL-15, serve to boost the protective responses to pathogens that are otherwise faintly stimulatory to CD4^+^ T cells, while preventing unwanted systemic stimulation of unrelated CD4^+^ T cells and thus autoimmunity. Yet, high concentrations of IL-15 can increase proliferation of CD4^+^ T cells independently of polyclonal or Ag-specific activation [75]. Such high doses of IL-15 might in fact reflect severe pathological conditions, such as in the case of the highly inflammatory synovium of patients with rheumatoid arthritis [15], [76], inducing T cell proliferation in the absence of concomitant TCR signals [75].

Our experiments using neutralizing anti-IL-2 mAbs, and IL-2^−/−^ mice further showed that the growth activity of IL-15 on TCR-stimulated CD8^+^ T cells was independent of IL-2. But, for CD4^+^ T cells, IL-15 depended completely on IL-2 during the initial phase of T cell activation. Later, three to five days after TCR stimulation, IL-15 enhanced the proliferative response of CD4^+^ T cells to TCR stimulation independently of the presence of IL-2. This differential dependency on IL-2 could reflect a physiological situation in which innate immune cell-derived IL-15 only supports T cell expansion when IL-2 is produced by TCR-stimulated T cells or by antigen-presenting cells, such as DCs [77]. Later, IL-15 produced by activated monocytes/macrophages within inflamed tissue might support T cell expansion without a strict requirement for TCR signals and IL-2. Mechanistically, this switch from an early, IL-2 dependent growth activity to a late, IL-2 independent growth modus may result from the gradual acquisition by the TCR-stimulated CD4^+^ T cells of the high-affinity IL-15 receptor. Gene expression analysis indeed showed that activation of CD4^+^ T cells upregulated the high-affinity receptor subunit IL-15Rα. IL-15Rα can, in cis or in trans [78], combine with IL-2/15Rβ and the γ_c_ chain to form the heterotrimeric IL-15 receptor, promoting signaling in response to low doses of IL-15.

Interestingly, low-dose anti-CD3 stimulation of murine CD4^+^ T cells yielded CD25^−^ and CD25^Int^, but also CD25^High^ CD4^+^ T cells. These CD25^High^ CD4^+^ T cells persisted as a distinct population in the T cell culture and represent functional, activated descendants of naturally occurring CD25^+^ CD4^+^ Treg, as determined by depletion experiments, cross-over labeling and suppressor assays. Supplementation of IL-2 or IL-15 allowed conventional CD4^+^ T cells to proliferate in the presence of CD25^High^ Treg. IL-2 is a known growth/survival factor for Treg [79] that can enhance Treg functionality in vivo [61], [62], [74]. Also, Tregs can scavenge IL-2 thereby depriving Teff of growth factor and causing cell death [80]. Supplementation with IL-2, and as shown here also IL-15, can compensate for this lack of growth factor and explain the reduced suppression and/or increased proliferation in cytokine-supplemented Treg:Teff cocultures. Additionally, cultures that were depleted of nTreg in advance contain ample endogenous IL-2, possibly explaining the lack of effect of IL-15 supplementation in these cultures. As such, our data are in line with a recent report suggesting that IL-15 renders human Teff resistant to suppression by Treg [65].

This explicit growth promoting effect of IL-15 might be restricted to specific conditions as used in our T cell culture assays, namely low-dose IL-15 and suboptimal TCR occupancy. In the organism, such conditions may occur under certain circumstances, such as low level reactivity to self-antigens or chronic infection. In autoimmunity, IL 15 is detectable at heightened concentrations in the synovial fluid of rheumatoid arthritis patients [15], [16], the serum of patients with systemic lupus erythematosus [17] or type 1 diabetes [18], and the affected tissues in autoimmune thyroid disease [19] and celiac disease [20]. In infectious settings, IL-15 derived from innate immune cells can overcome the activation threshold formed by Treg, thereby linking the innate and the adaptive immune system to promote stronger T cell responses to foreign antigens. However, chronic infection can result in prolonged and/or heightened IL-15 expression which in turn, at unusual convergence of events, might link infection to development of autoimmune diseases.

Exploitation of the growth promoting activities of IL-15 supplementation is currently, after encouraging results in preclinical models [21]–[27], validated in clinical trials for tumor therapy and HIV vaccines. Nevertheless, our data showing that IL-15 can help Teff escape the suppression by Treg indicate it is advisable to monitor for autoimmune disease development when using IL-15.

## Materials and Methods

### Ethics Statement

The study and experimental protocols were performed according to the guidelines of and approved by the Ethical Committee on Laboratory Animal Experimentation of Ghent University.

### Mice

Female C57Bl/6N (H-2^b^) and BALB/c (H-2^d^) mice were purchased from Charles River (Iffa Credo, Italy). IL-2^−/−^ C57Bl/6 mice were kindly provided by A. Schimpl (University Würzburg, Germany). All mice were housed under specific pathogen-free conditions in temperature-controlled, air-conditioned facilities with 14/10 h light/dark cycles and food and water *ad libitum*. Spleens were harvested at 7–9 weeks of age.

### Reagents

Culture medium consisted of RPMI 1640 buffered with 12.5 mM HEPES (Life Technologies, Paisley, Scotland) and supplemented with 10% fetal bovine serum (FBS), 2 mM GlutaMAX-1 (Life Technologies), 100 U/ml penicillin, 100 µg/ml streptomycin, 1 mM sodium pyruvate, and 5×10–5 M β-mercaptoethanol. Human IL-15 was purchased from Peprotech (London, UK) and dissolved in PBS. Recombinant murine IL-2 and anti-mouse CD3 mAb (clone 145-2C11) were produced in-house. Soluble IL-15Rα T1 was used as IL-15 blocking agent, M4 as control protein [70]. FITC-conjugated anti-mouse CD4 (RM4-5), PE-conjugated anti-mouse CD25 (PC61), CyChrome-conjugated anti-mouse CD4 (RM4-5) or CD8 (53-6.7), biotinylated anti-mouse CD4 (RM4-5) or CD25 (PC61), anti-mouse IL2 blocking mAb (JES6-5H4 and S4B6), Fc-block (anti-mouse CD16/CD32, 2.4G2) were all from BD Biosciences (Erembodegem, Belgium). 5-(and-6)-carboxyfluorescein diacetate, succinimidyl ester (CFSE; Invitrogen) was dissolved at 1 mM in DMSO. Flow-Count fluorospheres for quantification of cell numbers were from Beckman Coulter (Paris, France). Protein transport inhibitor Brefeldin A and calcineurin inhibitor cyclosporin A (CsA) were from Sigma.

### Purification of T cell Subsets

Bulk splenocytes were processed in PBS, RBC were lysed using ammonium chloride buffer (3 min at room temperature), and cells were passed through a 40-µm cell strainer (BD Biosciences). T cell-depleted accessory cells (TdACs) were prepared from splenocytes by depletion of CD90^+^ (Thy1.2) cells using magnetic microbeads (Miltenyi, Bergisch Glabach, Germany). The column flow-through contained the T cell-depleted accessory cells (TdACs) and routinely showed less than 4% T cell contamination. TdACs were gamma-irradiated (50 Gy) before use.

CD25^high^ and CD25^Int^ CD4^+^ T cells were purified from activated CD4^+^ T cell cultures: First, CD4^+^ T cells were positively selected using biotinylated anti-CD4 mAb and streptavidin-conjugated microbeads according to the manufacturer’s instructions, typically yielding >95% CD4^+^ T cells. Next, cells were stimulated with 0.1 µg/ml anti-CD3 in the presence of irradiated TdACs (1∶5). After 3 days, cells were harvested, CD4 cells were enriched using CD4 microbeads (Miltenyi) and simultaneously stained with CyChrome-anti-CD4 and PE-anti-CD25 (2 µg/ml each) prior to flowcytometric sorting (FACSVantage, BD Biosciences). The CD25^high^ and CD25^intermediate^ fractions were sorted to >97% purity.

Negative selection of total CD4^+^ T cells was done using the Mouse CD4^+^ T Lymphocyte Enrichment Set - DM (BD Biosciences) according to the manufacturer’s instructions. Similarly, this kit was used for the isolation of CD25^−^CD4^+^ responder T cells, but with supplementation with a biotinylated anti-CD25 mAb (4 µg/ml).

### Cellular Activation and Proliferation Assays

2×10^5^ total spleen cells, or alternatively, 3×10^4^ CD4^+^ or CD8^+^ T cells together with 1.7×10^5^ irradiated TdACs, were seeded in flat bottom microwells with soluble anti-mouse CD3 mAb in the presence or absence of 3 ng/ml human IL-15, 400 nM CsA or 10 µg/ml blocking anti-mouse IL-2 mAb (clone JES6-5H4, data not shown for clone S4B6), as indicated. For the last 12 hours, 0.5 µCi ^3^H-thymidine was added to the cultures and cells were harvested on glass fiber filters to measure incorporated ^3^H-thymidine using a TopCount scintillation counter (Packard Instrument).

### Cell Staining and Absolute Cell Counting

Cells were washed twice with PBS, resuspended at 1×10^7^ cells/ml, and incubated with 1µM CFSE for 12 min at 37°C. The labeling reaction was stopped by addition of an equal volume of FCS, and cells were washed twice in medium. For cell counting, CFSE-labeled cells were recovered from micro-well cultures at the indicated time points. Cells were resuspended in FACS buffer (PBS, 0.5% FCS, 2 mM EDTA), stained for mouse CD4 and washed. Next, PI and a known quantity of beads were added, and the cells were acquired by flow cytometry. Absolute cell numbers were calculated using the following formula: Absolute number of CD4^+^ T cells = (number of beads added/number of beads counted) x number viable CD4^+^ T cells counted. Flowcytometric data collection from all stained samples was performed on a FACSCalibur using CellQuest software.

### Statistical Analysis

Statistical analysis was performed using GraphPad Prism (La Jolla, CA). Statistical significance was calculated by student t-test. Data were considered significantly different at p<0.05. ns = not significant, * *P*<0.05; ** *P*<0.01; *** *P*<0.001.
